# A convergence of global epidemics: diabetes as a modulator of neurodegenerative and neuro-inflammatory disorders

**DOI:** 10.3389/fneur.2026.1824840

**Published:** 2026-06-18

**Authors:** Louisa Leone, Tighe J. Kiernan, Satoshi Kuwabara, Michael Barnett, Emma Devenney, Rebekah M. Ahmed, Cindy Shin-Yi Lin

**Affiliations:** 1 Central Clinical School, Faculty of Medicine and Health, University of Sydney, Sydney, NSW, Australia; 2 The Brain and Mind Centre, University of Sydney, Sydney, NSW, Australia; 3 Department of Neurology, Chiba University Hospital, Chiba University, Chiba, Japan; 4 Department of Neurology, Royal Prince Alfred Hospital, Camperdown, NSW, Australia; 5 Neuroscience Research Australia (NeuRa), UNSW, Sydney, NSW, Australia

**Keywords:** dementia, diabetes mellitus, insulin resistance, neurodegeneration, neuroimmune disease, neuroinflammation, peripheral neuropathy

## Abstract

Diabetes mellitus (DM) and neurological disorders are rapidly converging global health burdens, driven by population ageing, the growing prevalence of metabolic syndrome, and limited early detection and disease-modifying therapies for many neurological syndromes. Beyond its established role in diabetes-related peripheral neuropathy, DM is increasingly implicated as a modifier of risk, phenotype, and prognosis across a wide range of central and peripheral nervous system diseases. In this narrative review, we synthesize current epidemiological, clinical, genetic, and mechanistic evidence examining the relationship between DM and 10 clinically important neurological disorders: Alzheimer’s disease (AD), vascular dementia (VaD), Parkinson’s disease (PD), Huntington’s disease (HD), amyotrophic lateral sclerosis (ALS), frontotemporal dementia (FTD), chronic inflammatory demyelinating polyradiculoneuropathy (CIDP), multiple sclerosis (MS), myasthenia gravis (MG), and neuromyelitis optica spectrum disorder (NMOSD). Across these conditions, DM acts as a context-dependent disease modifier, increasing risk in some disorders, appearing protective or delaying onset in others, and influencing disease phenotype, progression, and treatment response. We highlight potential areas of mechanistic convergence, such as insulin resistance, inflammation, disrupted energy homeostasis, and genetic predisposition, alongside important divergences shaped by disease-specific pathology. We also discuss the clinical and translational implications of this interface, including diagnostic challenges, opportunities for improved risk stratification, and growing interest in repurposing antidiabetic therapies, particularly metformin, glucagon-like peptide-1 receptor agonists, and sodium-glucose cotransporter-2 inhibitors, for neurological benefit. As the global burden of diabetes and neurological disease escalates, it is crucial to better understand the interplay between metabolic dysfunction, neurodegeneration, and neuro-immune pathways. The integration of insights across diseases may inform prevention strategies and support the development of therapeutic interventions at the metabolic-neurological interface.

## 1 Introduction

Diabetes mellitus (DM) is a complex metabolic disorder that poses an increasing global health challenge. It was estimated to affect 589 million adults worldwide in 2024, which is projected to rise to 853 million by 2050; associated costs exceed 1 trillion USD annually and are growing relentlessly (1). DM is broadly characterized by chronic hyperglycemia resulting from impaired insulin secretion, insulin action, or both (2). The two commonest subtypes of DM have distinct pathogeneses: Type 2 DM, which accounts for 90% of cases, is characterized by insulin resistance and relative insulin deficiency; Type 1 DM is caused by autoimmune destruction of pancreatic *β*-cells.

In parallel, disorders of the nervous system are collectively ranked as the leading cause of disability-adjusted life years in 2021, affecting over 3.4 billion individuals (3). Like DM, owing to an ageing population, the burden of neurological disease is rapidly increasing, compounded by limited progress in their prevention and management.

**Figure 1:**
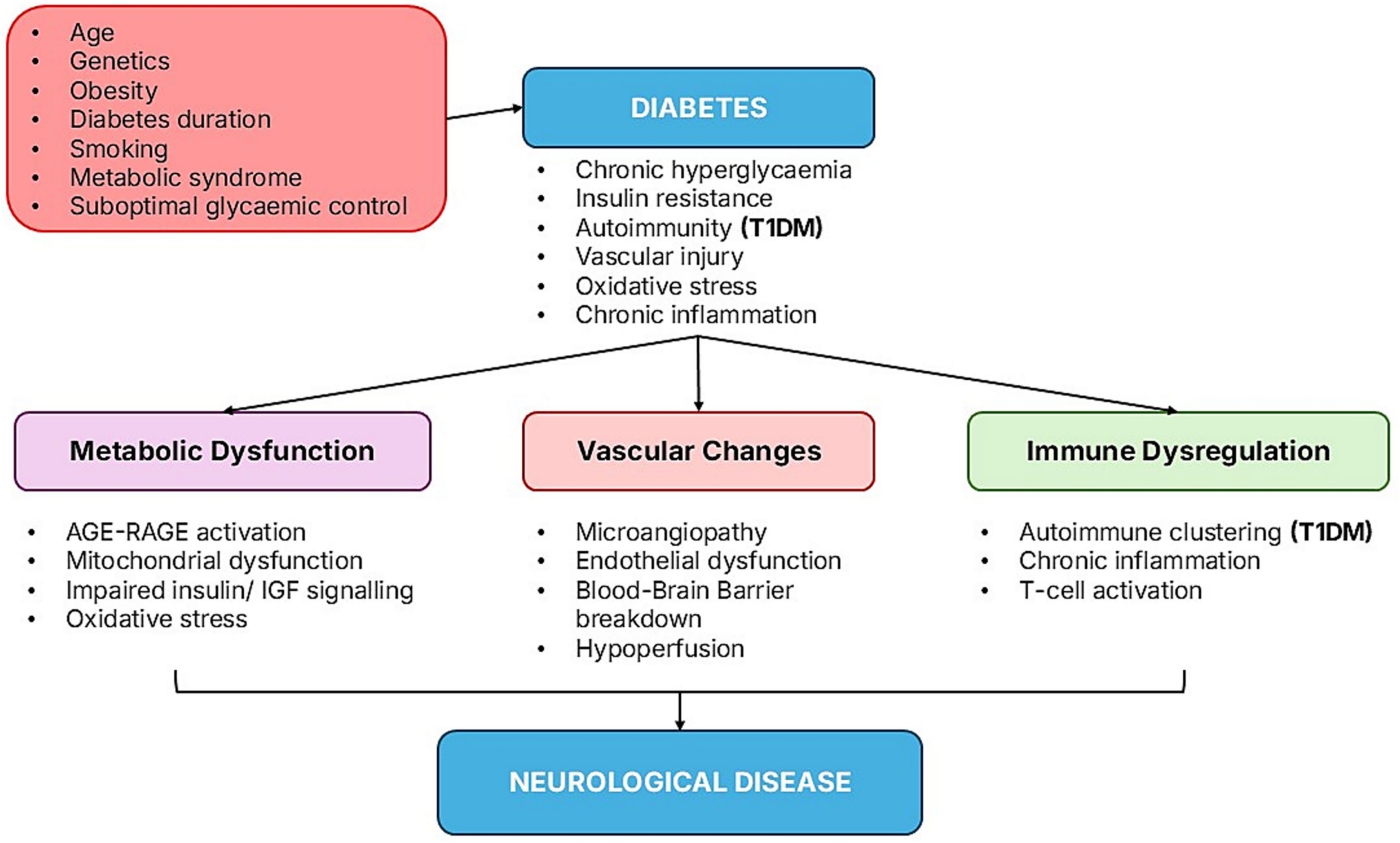
Intersection between DM and neurological disorders. Risk factors indicated in the red panel leading to “Diabetes” with a triad of pathophysiological pathways (metabolic dysfunction, vascular changes, immune dysregulation) converging on central and peripheral nervous system disease.

The connection between DM and the nervous system has been long recognized. Indeed, the most common complication of DM which affects approximately 50% of diagnosed individuals is diabetes-related peripheral neuropathy: a progressive symmetrical length-dependent sensorimotor neuropathy (4). The pathogenesis of nervous system involvement in DM is multifactorial, often involving features including abnormal protein aggregation, mitochondrial dysfunction, oxidative stress and inflammation (5). These mechanisms broadly fall into three interconnected categories: metabolic, vascular, and inflammatory, as seen in Figure 1. Persistent hyperglycemia creates a chronic pro-inflammatory milieu through the formation of advanced glycation end-products (AGE), which engage their receptors (RAGE) on neurons, glia, and the vascular endothelium, triggering oxidative stress, endothelial dysfunction, and microvascular injury (6). This AGE-RAGE interaction leads to nervous system hypoperfusion and may compromise the integrity of the blood–brain and blood-nerve barriers (7). In T2DM, insulin resistance contributes to altered lipid metabolism, mitochondrial dysfunction, and cytokine dysregulation - processes that collectively impair neuronal energy homeostasis and synaptic signaling (8). G-protein coupled receptor signaling can both promote and suppress neuroinflammation and subsequent neurodegeneration, highlighting. Type 1 DM, conversely, has been reported to coexist with other autoimmune disorders (e.g., thyroid disease, coeliac disease, and pernicious anemia) (9). This immunological overlap provides a plausible link to inflammatory and demyelinating nervous system disorders.

Owing to these intersecting metabolic, vascular, and inflammatory mechanisms, a growing body of evidence has linked DM to various central and peripheral nervous system diseases. The association between diabetes and cognitive decline is the most well-established, ranging from mild cognitive impairment to dementia. Diabetes has been most consistently associated with an increased risk of Alzheimer’s disease, Parkinson’s disease and inflammatory peripheral neuropathies (10–12). Interestingly, a neuroprotective role of DM and metabolic syndrome has been reported in amyotrophic lateral sclerosis, with purported mechanisms including lipid metabolic pathways which counteract the disease’s hypermetabolic state (13). Other important neurological conditions, notably Huntington’s disease, frontotemporal dementia, multiple sclerosis, and myasthenia gravis, have been variably linked to diabetes, though evidence remains unsynthesized. This underscores the complexity of diabetes-associated neurobiology and the need for a comparative framework that integrates findings across diverse disease categories.

Accordingly, this Review critically synthesizes current evidence linking DM to 10 clinically and epidemiologically important chronic neurodegenerative and neuroinflammatory disorders spanning central and peripheral nervous system pathology: Alzheimer’s disease (AD), vascular dementia, Parkinson’s disease (PD), Huntington’s disease (HD), amyotrophic lateral sclerosis (ALS), frontotemporal dementia (FTD), chronic inflammatory demyelinating polyradiculoneuropathy (CIDP), multiple sclerosis (MS), myasthenia gravis (MG), and neuromyelitis optica spectrum disorder (NMOSD). These conditions were chosen as they have established or emerging literature on their relationship with DM. For each, we summarize epidemiological and clinical relationships, evaluate mechanistic convergence and divergence, and highlight translational implications including prevention strategies, diagnostic challenges, biomarker opportunities, and the emerging potential to repurpose antidiabetic therapies for neurological benefit.

This integrated synthesis is timely given the simultaneous expansion of diabetes prevalence and neurological disease burden, and the growing opportunity to translate mechanistic insights into disease-modifying interventions at the metabolic-neurological interface.

## 2 Diabetes and neurological diseases

### 2.1 Neurodegenerative diseases

#### 2.1.1 Alzheimer’s disease

Alzheimer’s disease (AD) is the most common cause of dementia worldwide, with a global prevalence exceeding 50 million individuals aged over 60 in 2021, and projected to increase four-fold by 2040 (14) AD results in a progressive decline in cognition and episodic memory (15). Pathologically, AD is characterized by extra neuronal aggregations of beta amyloid (Aβ) plaques and intracellular tau proteins. Epidemiological studies in the US reveal that modifiable risk factors account for up to 40% of dementia cases, with diabetes cited as a principal risk factor (16).

From a clinical standpoint, chronic hyperglycemia and insulin resistance have been associated with decrements in cognition and memory that correlate with reduced temporal and frontal lobe volumes on imaging (17). Epidemiological data supports this biological overlap; the most comprehensive meta-analysis included over 1.7 million people from 17 prospective studies and reported a pooled relative risk of 1.53 (95% CI 1.42–1.63) for incident Alzheimer’s disease in people with Type 2 DM compared to those without Type 2 DM (18) These shared traits have led to the labelling of AD as “Type 3 Diabetes” (19).

There are several mechanistic metabolic links between DM and AD. Indeed, insulin and insulin-like growth factors (IGF-I and IGF-II) are widely expressed in the central nervous system and are crucial for regulating metabolism, neuroplasticity, and neurotransmitter release (15).

In T2DM, insulin resistance impairs these functions, contributing to the cognitive decline observed in AD. The accumulation of Aβ plaques, a pathological hallmark of AD, is also influenced by hyperinsulinaemia. A key factor in Aβ plaque accumulation is insulin-degrading enzyme (IDE), which degrades both insulin and Aβ. When insulin levels are elevated, IDE preferentially clears insulin, reducing Aβ clearance efficiency, which promotes plaque accumulation in the brain (20). Additionally, insulin resistance impairs insulin- IGF-I signaling in the CNS, which ordinarily controls the cleavage of amyloid precursor protein (APP). Derangement in these pathways leads to a transition in APP cleavage from non-amyloidogenic breakdown to amyloidogenic breakdown, contributing to central Aβ accumulation through both reduced clearance and increased production of Aβ (21, 22). Furthermore, both AD and T2DM share mitochondrial dysfunction and oxidative stress as key pathophysiological features, particularly affecting the hippocampus and frontal cortex-regions which are critical for memory (23).

Cerebral small vessel disease, a known diabetes macrovascular complication, is also increasingly appreciated as a driver of cognitive impairment and dementia. Vascular pathology both triggers and accelerates neurodegeneration in a vicious cycle of cerebral hypoperfusion and oxidative stress (24). Indeed, the apolipoprotein E ε4 (APOE ε4) polymorphism, which affects cholesterol transport, is the strongest genetic risk factor for AD, purportedly linked to its ability to induce endothelial dysfunction. A recent cohort study involving over 100000 participants showed that those harboring a combination of T2D and APOE ε4 had the highest risk of AD (HR 2.87) when compared to age-matched controls, exceeding those with T2D alone (HR: 2.06) and APOE ε4 alone (HR 1.35) (25). This suggests a cumulative effect that is consistent with a broader link between metabolic and vascular dysfunction and AD.

The evidence-base for T1DM is smaller albeit growing, and indeed a recent 2025 systematic review and meta-analysis of six studies found a pooled hazard ratio of 1.50 (95% CI 1.25–1.80) for all-cause dementia in patients with T1DM (26). Notably, younger age at diabetes diagnosis appears to confer greater dementia risk in both diabetes types (27). Though not traditionally associated with insulin resistance, alternative mechanisms have been proposed underscoring this relationship, including chronic hyperglycemia, severe hypoglycemia, microvascular and macrovascular complications (28).

Together, these findings establish that DM - via convergent metabolic, vascular, and inflammatory mechanisms–is associated with increased susceptibility to AD. While evidence is strongest for T2DM, emerging data in T1DM suggest that a pro-inflammatory environment and chronic glycemic instability may elevate the long-term risk of neurodegeneration. Defining the relative contributions of these pathways will be essential for improving risk stratification and guiding targeted prevention.

#### 2.1.2 Vascular dementia

Vascular dementia (VaD) is a form of dementia that develops through cerebrovascular disease or cerebrovascular accidents resulting in brain infarction. This leads to a sudden drop in cognitive function following a cerebrovascular event or gradual deterioration due to cerebrovascular small vessel disease in broad cognitive domains, including executive function, processing speed, and attention (29). VaD is the second most common cause of dementia after AD, though estimates of its prevalence vary considerably depending on diagnostic criteria and whether mixed pathology is included. A recent review by Smith and colleagues cited that prevalence of VaD in those with diabetes ranges from 8.9–49.7%; mixed pathology is more common than isolated VaD (30).

Across phenotypes, VaD is the dementia subtype that has been most strongly associated with T2DM, above AD, owing to comorbid cardiovascular and cerebrovascular disease which contributes to vascular-mediated brain injury (31). A 2013 meta-analysis of prospective observational studies reported a pooled RR of 2.27 for VaD in individuals with T2DM (95% CI 1.94–2.66) (32). However, one confounder is that VaD and mixed dementia are often conflated in older cohorts, confounding estimates of its prevalence. Interestingly, a 2023 review found that the use of anti-diabetic medication, particularly metformin, was associated with a reduced risk of developing VaD, suggesting that treatment of vascular risk factors may concomitantly address cognitive decline (17). This is explored in greater depth in Section 3.2.1. While age remains the most significant contributor to dementia onset, converging evidence supports a direct and compounding role of T2DM in exacerbating neurovascular injury and accelerating cognitive impairment.

#### 2.1.3 Parkinson’s disease

Parkinson’s disease (PD) is the second most common neurodegenerative disorder globally following dementia, with diagnosis rates doubling since 1990 and predicted to rise further over the next 30 years (33). PD has a multifactorial aetiology and clinically presents as a movement disorder characterized by tremor, rigidity, bradykinesia and postural instability, along with nonmotor features such as cognitive impairment, autonomic instability, and mood disturbance (34). It is pathologically characterized by the deposition of alpha-synuclein aggregates, known as Lewy bodies, in the substantia nigra, along with mitochondrial dysfunction, altered intracellular trafficking, and neuroinflammation (35).

Comorbidities are increasingly recognized as important modifiers of the development and progression of PD, of which T2DM is notably significant. Indeed, T2DM is associated with more severe and accelerated motor symptoms, greater gait dysfunction, increased loss of independence (OR 2.08, 95% CI 1.34–3.25) and poorer mood outcomes in PD patients (36). Meta-analyses and large-scale cohort studies highlight an epidemiological link between T2DM and increased PD risk. Most recently, a 2023 systematic review by Aune et al. of 15 cohort studies with 29.9 million participants reported that diabetes was associated with a 27% increased relative risk of developing PD (RR 1.27, 95% CI 1.20–1.35) (37). This association was consistent across geographic regions, by sex, and across multiple subgroup and sensitivity analyses. This suggests that diabetes is a risk factor for developing PD, and its presence exacerbates PD severity and accelerates disease progression. These shared biological pathways have generated interest in repurposing antidiabetic medications for PD, with early studies suggesting potential clinical benefits (38). This will be further explored in Section 3.

Several overlapping pathophysiological mechanisms have been proposed to underpin the relationship between PD and T2DM, including insulin resistance, mitochondrial dysfunction, oxidative stress, chronic inflammation, and pathological protein aggregation (33). Dysregulated amylin homeostasis may represent one such mechanistic link. Amylin, a pancreatic peptide co-secreted with insulin, may be elevated during early insulin resistant states and is capable of forming amyloidogenic aggregates a capable of forming aggregates which have been implicated in neuroinflammation, oxidative stress and mitochondrial dysfunction, providing a potential mechanistic link between T2DM and neurodegenerative disorders including PD, along with AD (33). However, the link between pathology and clinical phenotype remains incompletely understood; for instance, a neuropathological study among 25 PD patients with T2DM indicated that those with DM had more aggressive disease, independent of neuropathological findings (39).

Interpretation of the relationship between PD and DM is also complicated by the substantial clinical and pathological overlap between PD and related neurodegenerative disorders. For example, cognitive impairment is a core nonmotor feature of PD, with many patients ultimately developing Parkinson’s disease dementia, while dementia with Lewy bodies shares overlapping *α*-synuclein pathology and clinical manifestations (40, 41). Consequently, observed associations with DM may vary across epidemiological studies depending on diagnostic definitions, and inclusion and exclusion criteria. Also, mixed PD, AD, and cerebrovascular pathology is common in older adults (42, 43). This exacerbates the challenge faced in isolating the relationship between PD and DM, potentially contributing to inconsistencies between epidemiological and neuropathological studies.

Overall, current evidence supports an association between DM and PD at both epidemiological and mechanistic levels, with diabetes additionally linked to more severe clinical phenotypes in established PD. However, the extent to which DM directly contributes to PD pathogenesis, and whether the association is bidirectional, remains uncertain. Future longitudinal studies with well-defined clinical and neuropathological criteria will be important in clarifying this relationship.

#### 2.1.4 Huntington’s disease

Huntington’s disease (HD) is a progressive autosomal dominant neurodegenerative disorder caused by an expanded CAG trinucleotide repeat in the huntingtin (HTT) gene on chromosome 4 (44). The resultant mutant huntingtin protein induces widespread neuronal dysfunction and death, particularly affecting the medium spiny neurons of the striatum. Clinically, HD typically manifests in mid-adulthood (ages 30–50) with progressive extrapyramidal motor symptoms, cognitive decline, and psychiatric disturbances (45).

Existing evidence suggests a potential link between diabetes mellitus (DM) and HD, particularly in animal studies, though clinical findings remain limited and heterogeneous (46). Early work by Farrer (47) reported a higher age-standardized prevalence of diabetes among 288 US-based Caucasian individuals with HD compared to controls, with first-degree relatives of HD patients being seven times more likely to develop DM than those without a family history of the disease. Subsequent small-scale studies have supported this association: one observed a dose-dependent relationship between the number of CAG repeats and insulin sensitivity measured via indirect calorimetry (48), while another involving 29 normoglycaemic HD patients demonstrated reduced insulin secretion capacity, decreased insulin sensitivity, and increased insulin resistance relative to controls 49. Conversely, Boesgaard and colleagues found no significant differences in glucose tolerance (assessed by oral glucose tolerance testing) between individuals with and without HD, although they noted that altered glucose metabolism may emerge in end-stage or juvenile-onset disease (50).

Mechanistically, mutant huntingtin appears to interfere with insulin signaling within pancreatic *β*-cells by sequestering insulin receptor substrate 2 (IRS-2) into protein aggregates, thereby impairing the PI3K/AKT/FoxO1 pathway (51). This results in diminished insulin secretion and increased β-cell apoptosis. Additionally, HD has been associated with hypothalamic and endocrine dysregulation, including elevated levels of insulin counter-regulatory hormones such as growth hormone and cortisol (52). As Szablewski (2025) notes, the directionality of this relationship remains unclear: while HD is characterized by altered glucose metabolism, mitochondrial dysfunction, and oxidative stress within the nervous system, it is uncertain whether these metabolic disturbances are a cause or a consequence of neurodegeneration (53).

#### 2.1.5 Amyotrophic lateral sclerosis

Amyotrophic lateral sclerosis (ALS) is a fatal neurodegenerative disease primarily involving the progressive degeneration of upper and lower motor neurons, typically resulting in death within 2 to 5 years from symptom onset (54, 55). Although its precise aetiology remains unknown, ALS is considered to arise from a complex interplay of genetic and environmental factors (56, 57). Hypermetabolism, defined by a rise in resting energy expenditure, is a common clinical feature of ALS, that has been linked to more aggressive disease (58). Therefore, the relationship between ALS and DM is potentially unique, where associated hyperglycemia, hyperinsulinaemia and increased adiposity (especially in T2DM) may buffer against ALS-associated catabolism (59).

Consistent with this hypothesis, initial epidemiological findings suggested an inverse association between T2DM and ALS risk, with several large population-based studies reporting reduced odds of ALS among individuals with T2DM. However emerging research has provided conflicting results. A 2021 systematic review by Vasta et al. encompassing 35 studies found that T2DM was associated with a lower risk of developing ALS, with OR = 0.47 in U. S.-based studies (95% CI 0.38–0.58) (60). Other included studies reported that DM may only protect against ALS at younger ages; for instance, one 2015 Swedish population-based case–control study by Mariosa and colleagues found that DM was protective against ALS in subjects aged over 70 years (OR 0.71, 95% CI 0.57–0.89 in the 70–79 age group and OR 0.56, 95% CI 0.40–0.78 in the > 80 age group), whereas it was associated with an increased risk of ALS in those under 50 (OR 3.15, 95% CI 1.40–7.08) (61). A possible explanation is that DM in younger individuals is likely to be T1DM, whereas that in older cohorts is T2DM. Indeed, Mariosa and colleagues specifically found that non-insulin-dependent diabetes showed a protective association (OR 0.66, 95% CI 0.53–0.81), while insulin-dependent diabetes showed no significant protective effect (OR 0.83, 95% CI 0.60–1.15) overall (61).

The relationship between ALS and T2DM is contradictory. In their systematic review and meta-analysis, Vasta and colleagues further noted that although T2DM was associated with delayed ALS onset, it did not appear to improve survival once ALS developed. Similarly, Martinelli et al. (2025) found that ALS patients with T2DM (“diALS”) exhibited more severe respiratory dysfunction and poorer scores on the ALS Functional Rating Scale-Revised (ALSFRS-R) at diagnosis, despite having protective features such as higher BMI and dyslipidaemia - factors generally linked to slower ALS progression (62). The study further reported that diALS patients experienced faster disease progression, higher likelihood of respiratory failure and marked weight loss, suggesting a paradox in which T2DM may delay ALS onset but contribute to more aggressive disease once ALS manifests.

Genetic studies have added further complexity. Using a two-sample Mendelian randomization approach, Zhang et al. (63) examined whether genetically predicted T2DM was causally linked to ALS. Interestingly, they found that in European populations, the OR of ALS per 1 SD increase in T2D was 0.96 (95% CI 0.92–0.996); in East Asian populations, this OR = 0.83 (95% CI 0.70–0.992). These researchers additionally discuss the heterogeneity of findings across populations and study designs suggests that this relationship may be modulated by population-specific factors, such as metabolic profiles, environmental exposures, or genetic variants.

There is also increasing evidence for dysregulation in CNS pathways involved in energy homeostasis, that may help reconcile the contradictory relationship between ALS and metabolic health. Indeed, the hypothalamus, which is a key CNS regulator of energy balance and body weight, has been implicated as a site of pathological involvement in ALS. A 2025 review by Chang and colleagues synthesized neuroimaging, neuropathological, and neuroendocrine data which demonstrated hypothalamic atrophy, neuronal loss, and TDP-43 pathology in ALS, with specific involvement of the hypothalamic nuclei governing feeding behavior and metabolic regulation (64). These changes have been associated with lower BMI, accelerated weight loss, and poorer survival, independent of motor neuron burden. Therefore, though the hyperglycemia, hyperinsulinemia and increased adiposity characteristic of T2DM may buffer against ALS-associated hypermetabolism, it is plausible that progressive hypothalamic pathology overrides these peripheral metabolic effects once ALS develops. This hypothesis warrants further investigation.

In summary, the current evidence suggests that T2DM is associated with a reduced risk of ALS and delayed disease onset but does not appear to confer benefit once ALS is established. Instead, comorbid T2DM may be associated with faster functional decline, suggesting that peripheral metabolic advantages are outweighed by disease-related catabolic processes and the broader vulnerability conferred by DM as a comorbid disease during later stages. Improved characterization of the central and peripheral metabolic pathways linking diabetes and ALS may help resolve this heterogeneity and inform the development of targeted metabolic, nutritional, and therapeutic strategies aimed at modifying disease progression in ALS.

#### 2.1.6 Frontotemporal dementia

Frontotemporal dementia (FTD) is the third most common dementia overall, affecting over 6 million individuals globally, and a leading cause of early-onset dementia (before age 65) (65). The disorder encompasses several clinical variants, the two commonest being behavioral variant FTD characterized by personality changes, executive dysfunction, and disinhibition; and primary progressive aphasia with nonfluent/agrammatic and semantic variants presenting with progressive language impairment. FTD is increasingly recognized as existing on a continuum with ALS, with 10–15% of patients with ALS meeting criteria for FTD at baseline, and, in one study, 30–40% of those with antecedent FTD having clinical or neurophysiological motor system dysfunction (65, 66).

This clinical heterogeneity closely mirrors the underlying proteinopathies that define FTD. Neuropathologically, FTD is characterized by focal degeneration of the frontal and temporal lobes and is classified primarily according to the dominant aggregated protein species: FTD–TDP, associated with TAR DNA-binding protein 43 (TDP-43) pathology and accounting for approximately 50% of cases, and FTD–tau, associated with abnormal aggregation of the microtubule-associated protein tau, comprising approximately 45% of cases (67).

In contrast to the associations between DM and AD and VaD, no epidemiological evidence exists in support of DM as a risk factor for FTD. A key explanation for this divergence lies in the greater heritable contribution to FTD pathogenesis: about 30–50% of cases of FTD are familial, reflecting a stronger genetic preponderance than AD and VaD, where metabolic and vascular risk factors exert a more prominent upstream influence (68). Nevertheless, important metabolic abnormalities are evident in FTD. Impaired cerebral glucose utilization is a hallmark feature across all FTD subtypes, in both patients and in preclinical models of the underlying proteinopathies (69). These finding may suggest that disrupted neuronal energy metabolism is a downstream consequence of primary neurodegenerative processes, rather than a driver initiated by systemic metabolic disease.

Evidence for systemic metabolic dysregulation in FTD is more limited but nonetheless intriguing and derives largely from small observational studies. For instance, in one cross-sectional study by Ahmed and colleagues involving a cohort of 90 patients with dementia (61 of which had FTD), those with FTD had increased triglyceride and insulin levels than healthy controls, suggesting a state of peripheral insulin resistance (70). As proposed by the authors, while insulin resistance may confer a survival advantage in motor neuron disease, similar metabolic states could exacerbate neurodegeneration in FTD by further compromising neuronal energy homeostasis. These observations highlight the possibility that metabolic factors act as disease modifiers in FTD.

Overall, current evidence suggests that the diabetes-FTD relationship is distinct from that observed in Alzheimer’s disease and vascular dementia. Metabolic dysfunction in FTD may represent a secondary or modifying process within a disease framework dominated by genetic and proteinopathic drivers. Clarifying how systemic metabolic states interact with genetically mediated neurodegeneration remains an important avenue for future research and may help refine disease-specific models of the diabetes-neurology interface.

### 2.2 Inflammatory nervous system diseases

#### 2.2.1 Chronic inflammatory demyelinating polyneuropathy

Chronic inflammatory demyelinating polyradiculoneuropathy (CIDP) is a rare and heterogeneous autoimmune-mediated peripheral neuropathy. Its typical form is characterized by symmetric proximal and distal weakness with sensory loss, evolving over at least 8 weeks. First-line therapy includes intravenous immunoglobulin (IVIG), corticosteroids, and plasma exchange, with subcutaneous immunoglobulin increasingly used.

Chronic inflammatory demyelinating polyradiculoneuropathy shares overlapping pathophysiological pathways with DM. Chronic hyperglycaemia induces microvascular dysfunction, oxidative stress, and low-grade inflammation, which collectively may disrupt immune tolerance and potentiate autoimmune demyelination (10). Despite this biological rationale, the association of CIDP and diabetes mellitus remains controversial. Early studies showed no association between CIDP and DM. Indeed, a 2009 population-based retrospective cohort study of over 100000 individuals identified 23 cases of CIDP, with only one (4%) having DM, compared to 12% of age- and sex-matched controls (71). Similarly, an Italian series found DM in 9% of 155 CIDP patients, matching the expected population prevalence (72). In contrast, later studies reported higher co-occurrence of DM and CIDP. A 2016 U.S. insurance claims analysis found a 9-fold higher prevalence of CIDP among those with diabetes vs. those without diabetes (73). European registry studies and a single-center series from Japan subsequently described a more modest, two-fold excess risk, with DM present in 14–19% of CIDP cases (74–76).

This controversy reflects the heterogeneity of diagnostic criteria for CIDP and the substantial clinical and electrodiagnostic overlap between CIDP and diabetes-related polyneuropathy. Earlier studies which showed no association between DM and CIDP used the American Academy of Neurology (AAN) and preceding Dyck criteria, which are highly specific but less sensitive for CIDP, requiring stringent electrophysiological confirmation of demyelination (77). This approach risks underdiagnosing CIDP in people with diabetes, where demyelinating features may be subtler or masked by coexistent axonal loss in diabetes-related polyneuropathy. Later studies employed the more sensitive European Federation of Neurological Societies/Peripheral Nerve Society (EFNS/PNS) criteria, which integrate clinical, electrophysiological and supportive features. However, this may result in misclassifying diabetes-related polyneuropathy with demyelinating features – such as slowed conduction velocities, prolonged distal latencies, or conduction block - as CIDP (74, 78). Emerging tools including nerve ultrasound, composite diagnostic scores, and biomarkers of nodal/paranodal autoimmunity may improve diagnostic fidelity in this challenging overlap population (79).

It is also pertinent to consider the known role of corticosteroid therapy - a cornerstone of inflammatory and autoimmune disease treatment – in inducing DM in those with CIDP. Corticosteroids are known to induce insulin resistance and increase hepatic glucose output, with prolonged courses conferring the highest risk of glucocorticoid-induced diabetes (80). However, comparative data on the incidence of DM in CIDP patients treated with vs. without steroids is extremely limited; in part because they have been superseded by IVIg particularly in high-income nations. A 2018 retrospective multicenter study comparing corticosteroid regimens found that adverse events including DM were reported in 8% of patients; though DM was not separately reported (81). Another Italian retrospective analysis comparing steroids vs. IVIg and plasma exchange for CIDP suggested that 5 of 155 patients treated with steroids developed DM; not reported as a complication in other treatment groups (82). However, this was hampered by limited follow-up (24 months). Therefore, the modulating effect of corticosteroids on CIDP and diabetes is plausible but incompletely understood.

Diabetes also appears to modify the clinical phenotype and treatment response of CIDP. Patients with both conditions are known to experience more severe neuropathy, greater disability, and decreased quality of life, likely reflecting both additive axonal injury from diabetes-related neuropathy and delays in diagnosing CIDP (83). While CIDP is highly treatable with immunotherapy, a recent systematic review and meta-analysis showed that response rates are lower in those with diabetes (61% vs. 71% in idiopathic CIDP) (84). This treatment response is improved with a predominance of demyelination, and shorter disease duration. These findings emphasize the clinical importance of early recognition and aggressive treatment of CIDP in people with diabetes, as timely intervention can recover function that might otherwise be lost to misdiagnosis.

Taken together, the primary clinical implication is that in people with diabetes, new or rapidly progressive weakness may prompt consideration of CIDP rather than attribution to diabetic neuropathy alone, which is important since it necessitates specific treatment with timely immune-modulating therapy that can reverse disability. Early recognition and precise diagnostic distinction between metabolic and immune-mediated neuropathy remain pivotal to improving functional outcomes in this high-risk population.

#### 2.2.2 Multiple sclerosis

Multiple sclerosis (MS) is the most common CNS inflammatory disease, affecting more than 2.8 million people worldwide (85). Characterized by inflammatory demyelination and neurodegeneration, MS leads to progressive impairments in mobility, vision, sensation, sphincter function and cognition. Comorbidities are increasingly recognized as important modifiers of the MS disease course; the most prevalent include cardiovascular conditions (e.g., hypertension and dyslipidaemia) and neuropsychiatric disorders (e.g., anxiety and depression), which are linked to faster disability progression, increased symptom burden (e.g., fatigue), and decreased quality of life (86). Diabetes mellitus, with its associated functional limitations and clustering with vascular and neuropsychiatric comorbidities, represents a clinically significant comorbidity that may exacerbate the overall burden of MS.

Mechanistically, T2DM and MS may intersect by metabolic-immune pathways. A systematic review and meta-analysis of observational studies showed that insulin resistance and hyperinsulinaemia – pathophysiological hallmarks of T2DM - are more prevalent in people with MS compared to healthy controls, although included studies were heterogeneous (87). This aligns with a 2025 genome-wide Mendelian randomization study which demonstrated that genetic liability to T2DM and higher triglyceride-glucose index (a marker of insulin resistance) were causally associated with an increased risk of MS. This association was mediated by the phenotype of CD8 + cytotoxic T cells, suggesting that metabolic dysregulation may potentiate CNS autoimmunity. Despite these mechanistic links, epidemiological data on the relationship between T2DM and MS remains mixed. One large Taiwanese population-based cohort study reported that those with T2DM had a moderately increased risk of developing MS compared to age- and sex-matched controls over 9 years of follow-up (adjusted HR 1.44, 95% CI 1.08–1.94) (88). This risk ratio was highest in women aged ≤ 50 years (HR 2.16, 95% CI 1.02–4.59). By contrast, MS does not appear to substantially increase the risk of T2DM. A 2023 systematic review and meta-analysis of 19 studies found the pooled prevalence of T2DM in the MS population to be 5%, which is lower than the ~10% prevalence in the age-matched general population (89). A 2024 nationwide Danish cohort study of 13,376 patients with MS and 66,880 matched controls reported a similar cumulative incidence of (predominantly type 2) diabetes mellitus over a median 8.3 year follow up (6.5% vs. 7.3%, HR 0.98, CI 0.92–1.04) (90). These findings suggest that metabolic–immune crosstalk provides a plausible link between T2DM and MS, but their clinical co-occurrence remains infrequent and shaped by complex gene–environment interactions.

Epidemiological links are stronger between Type 1 Diabetes Mellitus and MS, albeit still inconsistent. A 2014 review concluded that those with T1DM have a three- to five-fold increased risk of developing MS compared to the general population, rising to 20-fold in women, and that first-degree relatives of patients with MS have a 40% increased risk of T1DM (91). Conversely, the aforementioned large Danish nationwide cohort study found that the risk of incident T1DM in those with MS was not significantly different from matched controls (HR 1.60, 95% CI 0.98–1.40) (90). One explanation is that T1DM is usually diagnosed in childhood or adolescence while MS presents in young adulthood, therefore, the temporal sequence favors observation of T1DM before MS. Mechanistic links are largely underpinned by shared autoimmunity: autoimmune diseases are known to cluster in individual patients, and indeed both T1DM and MS are T-cell-mediated diseases characterized by pathogenic T-helper 1 cell responses. Genomic studies have identified polymorphisms in immune-related genes that confer a risk of T1D and MS, including the interleukin-2 receptor *α* gene, and the CLEC16A gene which is involved in mitophagy regulation (92–94). However, puzzlingly, their primary human leukocyte antigen (HLA) risk haplotypes are distinct and in some populations mutually exclusive (95). Shared environmental risk factors, notably Vitamin D deficiency, have also been identified (91).

Despite uncertainty about the epidemiological overlap between MS and diabetes, their intersection carries important clinical implications. Diabetes can amplify functional limitations, compound neuropsychiatric symptoms, and increase vascular risk in people with MS, exacerbating disease burden. Shared end-organ involvement further accentuates disease burden, particularly in the visual system: MS-related pathology spanning optic neuritis, optic radiation involvement, and retinal inflammatory disease may intersect with diabetic microvascular injury, increasing the risk of visual impairment and complicating attribution of symptoms. Diabetes-related peripheral neuropathy may additionally impair mobility and sensory function, mimicking or exacerbating MS-related neurological deficits, while both conditions can independently contribute to sexual dysfunction through autonomic, vascular, and spinal cord-related mechanisms.

Multiple sclerosis treatments also have important documented considerations in patients with diabetes. High-dose corticosteroids, commonly used for acute MS relapse, can precipitate hyperglycemia or unmask latent diabetes – though, they are seldom used for long durations (96). By contrast, in the pre-clinical setting, sphingosine-1-phosphate (S1P) receptor modulators approved for MS (e.g., fingolimod, ozanimod, and Siponimod) have demonstrated potential metabolic benefits relevant for diabetes. They may reduce autoimmune *β*-cell injury (relevant to T1D) (97), improve insulin resistance and metabolic inflammation (T2D), attenuate diabetic nephropathy via S1P1-dependent endothelial and anti-fibrotic pathways (98), and stabilize the blood-retinal barrier in early diabetic retinopathy (99, 100). In contrast, diabetes is a recognized risk factor for fingolimod-associated macular oedema, necessitating careful baseline and early ophthalmic monitoring in this population (101). In addition, alemtuzumab has been associated with secondary autoimmune disease, including rare cases of autoimmune type 1 diabetes (102). Recognizing and optimally managing diabetes in individuals with MS therefore represents a tangible opportunity to reduce cumulative morbidity, individualize therapeutic decision-making, and improve long-term outcomes.

#### 2.2.3 Myasthenia gravis

Myasthenia gravis (MG) is a rare acquired autoimmune disease characterized by autoantibodies against components of the postsynaptic membrane of the neuromuscular junction (NMJ), most commonly the nicotinic acetylcholine receptor (AChR) (103). It is a fluctuant condition with a core clinical manifestation of fatigable muscle weakness that may affect ocular, bulbar, respiratory, and limb muscles. Respiratory involvement is particularly severe and may necessitate intensive care admission and reliance on respiratory support. Affecting 100–350 per million people worldwide, the global incidence and prevalence of MG has substantially risen, particularly in older adults (104). Its onset exhibits a bimodal peak: women are more commonly affected before age 40, while men have a higher proportion after age 50 (105). Treatment includes symptomatic treatment with acetylcholinesterase inhibitors, immunotherapy (including corticosteroids and immunosuppressants) and surgical options, with intravenous immunoglobulin and plasma exchange reserved for disease exacerbations. Adequate treatment typically stabilizes MG, and unlike other neuroimmune conditions like MS, it is not characterized by continuous disease progression (103).

Diabetes mellitus is a principal comorbidity faced by patients with MG, and increasing evidence supports that DM is overrepresented in those with MG. Indeed, a 2023 systematic review and meta-analysis of 11 studies comprising 16,825 patients showed that the pooled prevalence of DM among MG patients was 15.6% (95% CI 8.4–24.7%) (106). Compared to healthy controls, MG patients had a modestly increased risk of developing DM (RR = 1.24, 95% CI 1.01–1.54). However, the directionality and drivers of this association are unclear.

Several studies suggest that treatment-related factors may play a central role in the development of T2DM in MG. Interestingly, in support of MG as an antecedent for DM, another systematic review specifically identified an association between MG and an increased risk of gestational DM (OR = 1.56, 95% CI 1.26–1.93) (107). It is contentious whether MG per se or its treatment drives subsequent DM, particularly in the context of T2DM. For instance, retrospective cohort analysis using data from the Taiwan National Health Insurance Database identified that among 1520 patients with MG (compared to age- and sex-matched controls), MG patients without corticosteroid use had no increased risk of developing DM (HR = 1.05, 95% CI 0.79–1.40), whereas MG patients with corticosteroid use had a 1.46-fold increased risk of developing DM (HR = 1.46, 95% CI 1.15–1.86) (107). This alludes to the importance of judiciously using corticosteroids particularly in patients with MG and existing metabolic risk factors. The emergence of targeted biologic therapies such as complement inhibitors (e.g., eculizumab) may also have metabolic relevance in MG, particularly as steroid-sparing strategies may address glucocorticoid-associated hyperglycemia and diabetes risk (108).

Reduced physical activity secondary to fatigable muscle weakness may further contribute to metabolic risk. In a single-center cross-sectional study, Li and colleagues reported a higher prevalence of glucose and lipid metabolic abnormalities among 102 patients with MG who had not received corticosteroid (109). While causality cannot be inferred, these findings raise the possibility that disease-related reductions in physical activity may independently increase susceptibility to metabolic dysfunction, underscoring the importance of individualized exercise strategies where feasible in patients with MG.

In contrast, the association between MG and type 1 diabetes mellitus (T1DM) appears more consistent with shared autoimmune susceptibility. A recent genome-wide association study found evidence for a genetic link between MG and T1DM, among other autoimmune diseases, that extends beyond the HLA region alone (110). This may be particularly true for those with MG harboring AChR antibodies (seropositive MG); for instance, a retrospective single-center cohort study by Toth and colleagues found that among 109 patients with MG (66% seropositive), 13 (18%) seropositive MG patients carried a diagnosis of DM; 7 with T1DM and 6 with T2DM (111). Notably, T1DM preceded the diagnosis of MG in six of these seven cases, suggesting that pre-existing autoimmune dysregulation may contribute to MG pathogenesis in a subset of patients.

Preclinical evidence offers several additional mechanisms that may clarify the relationship between MG and DM. Hyperglycemia exacerbates MG by promoting the differentiation and activation of circulating T follicular helper cells (112). The AGE/RAGE signaling pathway may also provoke autoimmunity in MG, independent of hyperglycemia (113).

Overall, there is compelling observational evidence for a multifactorial association between MG and DM. For T2DM, though corticosteroid exposure and reduced physical activity are implicated, they are insufficient in fully explaining the link, suggesting shared inflammatory pathogenetic pathways. In contrast, the relationship between MG and T1DM is more consistent with shared autoimmune susceptibility, supported by genetic data. While preclinical studies indicate that metabolic dysregulation may influence immune mechanisms relevant to MG, further longitudinal and mechanistic studies are required to clarify causality and clinical significance.

#### 2.2.4 Neuromyelitis optica spectrum disorder (NMOSD)

Neuromyelitis optica spectrum disorder (NMOSD) is a rare, relapsing autoimmune inflammatory CNS disease that presents with optic neuritis, myelitis, and/or brainstem and encephalitic syndromes (114). The disease pathogenesis most commonly involves circulating serum antibodies against astrocytic aquaporin 4 (AQP4) channels, which mediate water transport across the cell membrane (herein termed seropositive NMOSD). Acute attacks are treated with high-dose glucocorticoids and plasma apheresis, while various immunomodulatory therapies are used for relapse prevention (115).

Like other neuroinflammatory conditions, the most plausible relationship between NMOSD and T1DM would be through an autoimmune diathesis. However, there is no epidemiological evidence to suggest that NMOSD clusters with T1DM as an autoimmune disease. At a genomic level, one recent genome-wide study identified a shared association between NMOSD and T1DM with a complement protein (C4A) genetic locus (116). However, the genetic risk profile of NMOSD has not been linked to T1DM.

The link between NMOSD and T2DM is stronger. Indeed, emerging evidence suggests that patients with NMOSD may be at increased risk of T2DM, particularly those managed with steroid medication. A 2024 case–control study using data from the Korean National Health Insurance Service database reported a 1.77-fold increased risk of developing T2DM among those with NMOSD treated with corticosteroids compared with healthy controls, however, this risk did not persist with those not receiving corticosteroids (117). This is consistent with the known diabetogenic effect of corticosteroids (outlined in 2.2.1), which may be particularly relevant in NMOSD where prolonged and high-dose corticosteroid therapy forms a cornerstone of treatment. In addition, a 2021 case–control study involving 56 patients with seropositive NMOSD demonstrated 2.26-fold higher odds of hyperinsulinaemia compared to healthy matched controls, though the prevalence of hyperglycaemia did not differ between groups (118). However, these findings are limited by small sample size.

The impact of DM on the incidence, course, and outcomes of NMOSD remain poorly characterized. One Japanese retrospective cohort study of 4231 individuals hospitalized with NMOSD suggested that those with existing diabetes had a higher risk of serious infection complicating admission (HR = 1.43, 95% CI 1.04–1.96) (119). This is consistent with the known immunosuppressive effects of DM; though not specific to NMOSD, it suggests DM is a clinically significant comorbidity. Meanwhile, a nationwide study from the United States reported that diabetes did not influence 30-day readmission rates among patients hospitalized with NMOSD, however, interpretation is limited by the short follow-up period (120).

Important clinical overlap may also exist between diabetes-related complications and NMOSD manifestations. For example, like MS, diabetes-related retinopathy may compound the effects of optic neuritis, while diabetes-related peripheral neuropathy and NMOSD-related transverse myelitis share overlapping sensory and motor impairments. Despite these potentially additive effects on neurological disability and quality of life, this area remains largely unexplored.

Mechanistically, NMOSD is associated with inflammatory mediators that may eventuate in insulin resistance. One emerging player is IL-6, which promotes the differentiation of plasmablasts that produce pathogenic AQP4-IgG antibodies, sustains the pro-inflammatory milieu that drives astrocytic injury, and is elevated in the cerebrospinal fluid and serum during acute attacks (121). Critically, IL-6 is also a well-established mediator of systemic insulin resistance, acting via JAK-STAT3 signaling to suppress insulin receptor substrate phosphorylation and promote hepatic glucose output (122). Consistent with this mechanistic link, the 2021 case–control study described above demonstrated a significant positive correlation between circulating insulin levels and interleukin 6 (IL-6) – however, causality remains uncertain and the study is limited by small sample size (118). This hypothesis gains translational relevance from the fact that IL-6 receptor blockade (satralizumab, tocilizumab) is now an approved therapeutic strategy in NMOSD (123), raising the untested but intriguing possibility that these agents may confer incidental metabolic benefit in patients with comorbid insulin resistance.

Overall, the relationship between NMOSD and diabetes remains incompletely understood but represents an emerging area of clinical and translational interest. Existing evidence suggests that corticosteroid exposure, systemic inflammation, and overlapping neurological morbidity may contribute to metabolic dysfunction in NMOSD. Longitudinal studies are needed to clarify the bidirectional relationship between NMOSD and diabetes, including the impact of glycemic status on disease activity, disability progression, and long-term neurological outcomes (Tables 1, 2).

**Table 1:** Summary of the overlap between DM and neurological disorders.

**Neurological disorder**	**Type 1 diabetes mellitus (T1DM)**	**Type 2 diabetes mellitus (T2DM)**	**Overall evidence base**	**Key references**
Neurodegenerative diseases				
Alzheimer’s disease (AD)	Increased risk of all-cause dementia (pooled HR 1.50, 95% CI 1.25–1.80); younger age at diabetes onset associated with greater cognitive risk	Increased risk of incident AD (pooled RR 1.53, 95% CI 1.42–1.63); additive risk observed with APOE ε4 carriage (HR 2.87)	✓✓✓	(17–28)
Vascular dementia (VaD)	Limited evidence for an independent association	Strong and consistent association with VaD (pooled RR 2.27, 95% CI 1.94–2.66); treatment of vascular risk factors, including metformin use, may reduce risk	✓✓✓	(17, 29–32)
Parkinson’s disease (PD)	Insufficient epidemiological evidence specific to T1DM	Increased PD risk (RR 1.27, 95% CI 1.20–1.35); associated with greater motor severity, gait dysfunction, functional decline, and accelerated progression	✓✓✓	(33–43)
Huntington’s disease (HD)	Limited evidence	Possible association with insulin resistance, impaired insulin secretion, and altered glucose metabolism; clinical evidence remains heterogeneous and inconclusive	✓	(44–53)
Amyotrophic lateral sclerosis (ALS)	No clear protective association identified	Several studies report reduced ALS risk and delayed onset in T2DM, though findings are heterogeneous; T2DM may be associated with more aggressive disease following ALS onset	✓✓	(54–64)
Frontotemporal dementia (FTD)	No established epidemiological association	No established epidemiological association; metabolic abnormalities may represent downstream or disease-modifying processes rather than causal drivers	✓	(65–70)
Neuroinflammatory diseases				
Chronic inflammatory demyelinating polyradiculoneuropathy (CIDP)	Not separately characterized	Increased CIDP prevalence reported in people with diabetes (approximately 2–9-fold depending on diagnostic criteria); associated with greater disability and lower treatment response rates	✓✓	(71–84)
Multiple sclerosis (MS)	Shared autoimmune susceptibility reported; epidemiological evidence inconsistent	Epidemiological evidence mixed; some studies report modestly increased MS risk (HR 1.44), whereas others demonstrate no significant association	✓✓	(85–102)
Myasthenia gravis (MG)	Association supported by shared autoimmune susceptibility and genetic overlap; T1DM may precede MG onset in some patients	DM overrepresented among patients with MG (pooled prevalence 15.6, 95% CI 8.4–24.7%); corticosteroid exposure associated with increased diabetes risk	✓✓	(103–113)
Neuromyelitis optica spectrum disorder (NMOSD)	No clear epidemiological association; evidence for shared autoimmune susceptibility limited to single gene locus	Increased T2DM risk reported in corticosteroid-treated patients (HR 1.77); hyperinsulinaemia more common in seropositive NMOSD	✓	(114–123)

RR = relative risk; OR = odds ratio; HR = hazard ratio; CI = confidence interval; NS = not significant. Overall evidence base: ✓✓✓ = strong and consistent epidemiological and/or mechanistic evidence supported by meta-analyses or large cohort studies; ✓✓ = moderate evidence with some heterogeneity or limited longitudinal data; ✓ = preliminary, inconsistent, or primarily mechanistic/small-cohort evidence.

**Table 2:** Mechanistic link between DM and neurological disorders.

**Mechanism/Pathway**	**AD**	**VaD**	**PD**	**HD**	**ALS**	**FTD**	**MS**	**CIDP**	**MG**	**NMOSD**
Metabolic dysregulation										
Insulin resistance and impaired signaling	✓✓	✓	✓✓	✓	Paradoxical^1^	✓	✓	✓	✓	✓
Hyperglycemia and AGEs/RAGE	✓✓	✓✓	✓	✓	✓ (protective)	–	–	✓	✓	-
Mitochondrial dysfunction	✓✓	✓	✓✓	✓✓	✓	–	–	✓	–	–
Vascular injury										
Endothelial dysfunction and BBB/BNB disruption	✓	✓✓	✓	–	–	–	✓	✓✓	–	–
Microvascular ischemia	✓	✓✓	✓	–	–	–	✓	✓	–	–
Protein aggregation and neurotoxicity										
Proteinopathy (e.g., Aβ/ tau/ α-synuclein/ amylin) disturbing neuronal metabolic homeostasis	✓✓	–	✓✓	✓	–	✓✓	–	–	–	–
IDE competition (insulin vs. Aβ)	✓✓	–	–	–	–	–	–	–	–	–
Inflammation and immunity										
Chronic neuroinflammation (cytokines, glia)	✓✓	✓	✓✓	✓	✓ (late)	–	✓✓	✓✓	✓	✓
Autoimmune overlap (T1DM-related)	–	–	–	–	–	–	✓✓	✓	✓✓	–
Other										
Oxidative stress	✓✓	✓	✓✓	✓✓	✓	–	✓	✓	–	–
Hypothalamic/endocrine dysregulation	–	–	–	✓✓	✓ (protective)		–	–	–	–

Summary of the principal mechanistic pathways proposed to underlie the association between DM and neurological disorders. Mechanisms are not mutually exclusive and frequently interact across metabolic, vascular, inflammatory, and neurodegenerative pathways. ✓✓ = strong/consistent evidence (multiple studies, meta-analyses); ✓ = moderate/emerging evidence; − = limited/weak/no evidence. 1: Protective association with delayed onset but potentially accelerated progression after disease onset.

## 3 The potential neuroprotective role of diabetes treatment

The intersecting biology of diabetes and neurological disease promotes important implications for prevention, risk stratification, and treatment of these comorbid conditions.

### 3.1 Lifestyle and glycemic control as neuroprotective strategies

Glycemic control remains the cornerstone for neuroprotection in diabetes. Near-normal glycemic management, implemented early in diabetes, effectively delays or prevents diabetes-related peripheral neuropathy, cardiovascular autonomic neuropathy in type 1 diabetes, with more modest benefits demonstrated in T2DM. Interestingly, one major trial (ACCORD MIND), showed that intensive glycemic management targeting HbA1c < 6.0% had no cognitive benefit compared to standard control (7.0–7.9%), suggesting that moderate targets may be optimal for neuroprotection (124). This is also supported by studies demonstrating the deleterious risks of hypoglycemia which dose-dependently increases the risk of neurological decline (125). Taken together, the effects of hyper- and hypoglycemia demonstrate a therapeutic challenge in diabetes and neurological disease, affirming the importance of euglycemia, rather than aggressive glucose-lowering, for neuroprotection.

Non-pharmacological interventions such as diet and exercise are a fundamental part of diabetes management and are both feasible and cost-effective. They show promising albeit modest neuroprotective benefits, being most researched in the context of cognitive impairment and dementia. Indeed, one large prospective study involving over 160,000 patients with diabetes showed that those with combined healthy lifestyle factors, defined via a lifestyle score of 7 (no smoking, moderate alcohol, regular physical activity, healthy diet, adequate sleep, less sedentary behavior, frequent social contact), had a 54% lower dementia risk compared to those with scores of 0–2, reducing their 10-year absolute risk of dementia from 5.22 to 1.72% (126). This association was independent of glycemic control and diabetes medication, emphasizing that lifestyle modification provides neuroprotection beyond glucose-lowering effects alone. Promising findings from the US POINTER randomized clinical trial showed that in over 2,000 older adults with sedentary lifestyles, suboptimal diets, and at least two metabolic risk factors, a 2-year, structured, high-intensity multidomain lifestyle intervention produced significantly greater improvements in global cognition than a lower-intensity, self-guided intervention (127). This emphasizes a potential therapeutic role of structured and intensive lifestyle modification.

Though evidence for other neurological syndromes remains sparse, mechanistically, lifestyle interventions target the major biological pathways linking diabetes to nervous system damage: improved peripheral and central insulin sensitivity, reduced neuroinflammation and mitochondrial dysfunction, enhanced cerebrovascular function and blood–brain barrier integrity, increased neurotrophic factor signaling, and reduced accumulation of advanced glycation end products (128). Clarifying the spectrum of diabetes-associated neurological disorders and their shared pathophysiology will be essential for guiding future research. Such work may reveal opportunities for simple, scalable, and low-risk nonpharmacological strategies capable of meaningfully modifying neurological outcomes in diabetes.

### 3.2 Repurposing antidiabetic medications for neurodegenerative pathways

There is growing interest in the neuroprotective actions of antidiabetic drugs beyond glycemic control, suggesting a potential role for repurposing these agents to modify neurological disease trajectories in diabetes. This is summarized in Table 3.

**Table 3:** Antidiabetic drug classes and their relationships with neurological disease.

Drug class (example agents)	Primary mechanisms	Preclinical evidence	Observational evidence	RCT evidence	Net assessment
Metformin	AMPK activation; suppresses hepatic gluconeogenesis; crosses blood–brain barrier; reduces Aβ, tau, and α-synuclein pathology; attenuates neuroinflammation	+++ Consistent neuroprotection across AD, PD, and HD models	++ Reduced VaD risk; lower ALS incidence (OR 0.83, 95% CI 0.75–0.93); modulation of MG–T2DM pathway	+ No dedicated neurological outcome RCTs; sparse clinical data	Promising
GLP-1 receptor agonists *(semaglutide, liraglutide)*	GLP-1R agonism; restores insulin–PI3K/Akt signaling; reduces Aβ, tau, and α-synuclein; enhances mitochondrial function; suppresses neuroinflammation	+++ Broad neuroprotection in AD, PD, and MS models; GLP-1R expressed in CNS	++ Reduced all-cause dementia risk (OR 0.55, 95% CI 0.35–0.86 in RCT meta-analysis); lower dementia incidence vs. comparators	+/− Evoke/Evoke+ (semaglutide): negative in established AD; liraglutide: slower cognitive decline without ADL benefit	Mixed
SGLT-2 inhibitors *(empagliflozin, dapagliflozin)*	Inhibits renal glucose reabsorption; enhances BDNF signaling; reduces neuroinflammation and protein hyperphosphorylation via improved systemic insulin sensitivity	++ Enhanced neurotrophic signaling; acetylcholinesterase inhibition; reduced tau hyperphosphorylation	+++ Reduced AD (aHR 0.81), VaD (aHR 0.69), and PD (aHR 0.80) risk in large cohort (*n* = 358,862); superior to incretin-based therapies in meta-analysis	+ No significant effect on dementia risk in dedicated RCT meta-analysis; most trials designed for cardiovascular endpoints with short follow-up	Promising
Insulin	Direct glucose lowering via insulin receptor activation; potential central neuroprotection only when plasma glucose is experimentally clamped	++ Neuroprotective under controlled glucose conditions; intranasal insulin models show cognitive benefit	− Increased dementia risk (HR 1.58, 95% CI 1.18–2.12); effect likely mediated by hypoglycemia-related oxidative stress and neuronal injury	+ GRADE trial: no significant cognitive differences vs. comparators at ~4 years in early, well-controlled T2DM	Caution
Sulfonylureas *(glimepiride, gliclazide)*	Stimulates pancreatic β-cell insulin secretion via KATP channel closure; peripheral glucose lowering without direct CNS activity	+ Limited neuroprotective data; some evidence of benefit in ALS models	+/− Increased dementia risk (OR 1.43, 95% CI 1.11–1.82); inverse association with ALS risk (OR 0.79, 95% CI 0.71–0.89)	+ GRADE trial: no significant cognitive differences in early, well-controlled T2DM	Mixed

Overall evidence base: +++, strong/consistent across multiple studies; ++, moderate/promising; +, limited/emerging; +/−, mixed or conflicting; −, negative or potentially harmful. Net assessment reflects the overall balance of evidence. Aβ, amyloid-beta; AD, Alzheimer’s disease; ADL, activities of daily living; aHR, adjusted hazard ratio; ALS, amyotrophic lateral sclerosis; AMPK, AMP-activated protein kinase; BDNF, brain-derived neurotrophic factor; CI, confidence interval; CNS, central nervous system; GLP-1R, glucagon-like peptide-1 receptor; GLP-1 RA, glucagon-like peptide-1 receptor agonist; HD, Huntington’s disease; HR, hazard ratio; KᴀTP, ATP-sensitive potassium channel; MG, myasthenia gravis; MS, multiple sclerosis; OR, odds ratio; PD, Parkinson’s disease; PI3K/Akt, phosphoinositide 3-kinase/protein kinase B; RCT, randomized controlled trial; SGLT-2, sodium–glucose cotransporter-2; T2DM, type 2 diabetes mellitus; VaD, vascular dementia; α-syn, alpha-synuclein.

#### 3.2.1 Metformin

Metformin, the first-line therapy for T2DM, lowers glucose by improving insulin sensitivity and suppressing hepatic gluconeogenesis, yet accumulating evidence suggests it may also exert direct neuroprotective effects. This has led to emerging interest in repurposing metformin as a potential neuroprotective therapy.

Preclinical models of AD, PD, and HD consistently show that metformin crosses the blood–brain barrier (BBB) and activates 5′AMP-activated protein kinase (AMPK)-dependent pathways that enhance mitochondrial bioenergetics, reduce Aβ, tau, and *α*-synuclein pathology, inhibit apoptosis, and attenuate neuroinflammation (129). Metformin also exerts effects relevant to VaD pathogenesis: it increases the integrity of the BBB by upregulating endothelial cell tight junctions and intercellular occludin and claudin proteins, primarily via AMPK-dependent pathways (130). However, observations from *in vivo* and in human studies remain mixed. Various mouse models report improved cognitive and motor outcomes with metformin (131), whereas others report negative effects including promotion of amyloidogenic processing, worsened tauopathy, and deleterious cognitive outcomes in certain preclinical models of dementia syndromes and in human cohorts (132). The factors underlying this heterogeneity remain incompletely understood. Broadly, current clinical evidence on the neurological impacts of metformin remain limited and largely confined to populations with existing diabetes, highlighting an important unmet research need in individuals without metabolic dysfunction (129).

Metformin has also been associated with a reduced risk of ALS in epidemiological studies. A 2021 systematic review and meta-analysis demonstrated that those with ALS were less likely to have been prescribed with metformin than controls (OR = 0.83, 95% CI 0.75–0.93) (133). However, this may represent an extension of the protective effect of diabetes itself on ALS onset.

Emerging preclinical evidence also implicates metformin as a modulator of the pathogenetic axis in T2DM and MG. A 2025 review by Al-Dhahi and colleagues proposed that metformin alleviates the pathophysiology of T2DM and MG by modulating the PI3K/AKT/mTOR/AMPK axis and promoting autophagy (134). This presents an additional opportunity for repurposing metformin in the neurological disease space.

#### 3.2.2 Glucagon-like peptide-1 receptor agonists

Glucagon-like peptide-1 receptor agonists (GLP-1RA) are a relatively new class of antidiabetic drug that mimic the action of GLP-1, an endogenous peptide secreted by enteroendocrine cells after food intake, to regulate insulin secretion, blood glucose levels, satiety, and body weight.

Recent evidence suggests GLP-1RA may play a neuroprotective role in diabetes-associated dementia. Indeed, a 2024 systematic review and meta-analysis of randomized clinical trials demonstrated that GLP-1RA were associated with a reduction in cognitive impairment and all-cause dementia in participants with diabetes when compared with placebo, usual care, or no glucose-lowering therapy (OR = 0.55, 95% CI 0.35–0.86) (135). However, this association did not hold when stratified by dementia subtype (AD and VaD). Animal studies suggest that GLP-1RA may restore insulin–PI3K/Akt signaling, reduce Aβ, tau, and *α*-synuclein pathology, enhance mitochondrial function and biogenesis, and suppress oxidative stress, apoptosis, and neuroinflammation across multiple neurodegenerative disease models (131).

However, the role of GLP-1RA in those with established dementia remains more ambiguous. In late 2025, the evoke and evoke+ Phase III randomized controlled trials (RCTs) showed that among patients with early-stage symptomatic AD, semaglutide failed to distinguish itself from placebo in its effects on AD progression (136). This led these trials to be discontinued. Comparatively, a phase 2b placebo-controlled trial in 204 patients with mild–moderate Alzheimer’s disease and no diabetes showed that those treated with liraglutide had a slower decline in cognitive function when measured by a specialized battery test, but there was no change compared to placebo in activities of daily living (137). Clearly, the evidence is contentious, and further research is needed to elucidate the timepoint at which GLP-1RA may benefit cognition and neurological outcomes, whether specific patient characteristics predict potential benefits, and whether this translates to a clinically meaningful difference in daily function.

There is also promising pre-clinical evidence for the therapeutic potential of GLP-1RA in CNS inflammatory disorders. Indeed, Gharagozloo and colleagues found that a novel GLP-1RA NLY01 delayed the onset and attenuated the severity of experimental autoimmune encephalomyelitis (a mouse model of MS) (138). Potential linked mechanisms were its ability to inhibit the activation and exocytosis of leukocytes, reduce the production of pro-inflammatory chemokines, and block the expression of genes associated with neurotoxic astrocytes in the optic nerves. Though in its infancy, if corroborated in human studies, it may represent a novel repurposing of GLP-1RA in the neurological setting.

#### 3.2.3 SGLT-2 inhibitors

SGLT-2 inhibitors are another new class of antidiabetic drugs that inhibit glucose reabsorption in the renal tubules. Preclinical models demonstrate that SGLT-2 inhibition enhances neurotrophic signaling (particularly via BDNF), modulates neurotransmission through acetylcholinesterase inhibition, and indirectly reduces protein hyperphosphorylation, aggregation, and neuroinflammation by improving systemic insulin resistance (131).

Compelling observational evidence supports that SGLT-2 inhibitors may reduce the risk of incident neurodegenerative diseases among patients with diabetes. A 2024 Korean study of 358,862 participants with T2DM aged ≥40 years showed that compared with other oral antidiabetic drugs, SGLT-2 inhibitors were associated with reduced risks of AD (adjusted hazard ratio [aHR] = 0.81, 95% CI 0.76–0.87), VaD (aHR = 0.69, 95% CI 0.60–0.78), and PD (aHR = 0.80, 95% CI 0.69–0.91). This was not affected by comorbidities, diabetes-related complications, and blood biochemical parameters (139).

Similarly, a 2025 target trial emulation study showed that, compared with other glucose-lowering drugs, patients initiating SGLT-2 inhibitors had a 43% lower incidence of AD and related dementias in a 9-year follow up period (HR = 0.57; 95% CI, 0.43–0.75). Notably, the same study observed a reduced incidence among GLP-1RA initiators (HR = 0.67; 95% CI, 0.47–0.96), and there was no difference between SGLT-2 inhibitors and GLP-1RA on the rate of incident dementia. In contrast, a systematic review and meta-analysis of nine studies reported that SGLT-2 inhibitors were associated with a significantly lower overall dementia risk compared with incretin-based therapies (including GLP-1RA; HR = 0.82, 95% CI 0.73–0.91), with empagliflozin demonstrating the most consistent protective association (140).

However, this is not corroborated by RCT data. A 2025 systematic review and meta-analysis of RCTs found no significant association between SGLT2 inhibitors and dementia risk when compared to placebo (135). This discrepancy between observational studies and RCTs likely reflects differences in study design, where observational studies remain vulnerable to residual confounding and treatment selection bias, while most RCTs of SGLT2 inhibitors were cardiovascular outcome studies with relatively short follow-up and without systematic assessment of cognitive endpoints. Therefore, given the promising yet observational nature of current evidence, RCTs specifically designed to evaluate cognitive outcomes are essential to confirm these findings and establish causality in the context of diabetes-associated neurological disease.

#### 3.2.4 Bridging the translational gap: lessons from GLP-1RA and SGLT-2i

The experiences with GLP-1RA and SGLT-2 inhibitors reveal a common pattern: robust preclinical neuroprotective mechanisms, encouraging but methodologically limited observational data, and inadequate or disappointing randomized trial evidence. This translational gap highlights several critical principles: (1) animal models may overestimate therapeutic potential or fail to capture human disease heterogeneity, (2) observational data cannot imply causation and may be influenced by confounding factors, and (3) prevention and treatment of neurodegeneration likely represent distinct therapeutic challenges with different optimal windows of intervention. Future efforts should emphasize well-structured RCTs with cognitive outcomes, prolonged follow-up, biomarker differentiation, and mechanistic examinations to conclusively determine the neuroprotective efficacy of these metabolic treatments.

#### 3.2.5 Limited benefit and potential harms of insulin and sulfonylureas

Insulin and sulfonylureas both lower blood glucose by increasing circulating insulin levels -insulin through direct replacement and sulfonylureas by stimulating pancreatic *β*-cell insulin secretion via closure of ATP-sensitive potassium channels. Contrasting the previous discussion, these drugs may be associated with increased dementia risk.

Although insulin has mechanistic neuroprotective potential, such effects are observed primarily when glucose levels are experimentally clamped. In routine clinical use, peripheral insulin therapy increases the risk of hypoglycemia, a well-established driver of oxidative stress, neuronal injury, and cognitive decline; accordingly, multiple cohort studies and meta-analyses link insulin use to higher dementia risk, including one pooled analysis reporting a 58% increased risk of new-onset dementia (HR = 1.58, 95% CI 1.18–2.12) (141). These findings suggest that the neurological harm associated with insulin reflects hypoglycemia-related metabolic instability rather than a direct neurotoxic effect of insulin itself.

Sulfonylureas similarly show neutral or adverse associations with cognitive outcomes. A 2023 network meta-analysis of 27 studies found sulfonylurea use associated with a 43% increased dementia risk (OR = 1.43, 95% CI 1.11–1.82), ranking second-worst after insulin among antidiabetic drug classes (142). However, evidence remains mixed: the 2025 GRADE randomized clinical trial found no significant differences in cognitive outcomes after ~4 years between glimepiride, insulin glargine, liraglutide, and sitagliptin in individuals with relatively early T2DM and well-controlled glycaemia (143). This suggests that in early-stage diabetes, when hypoglycemia burden is low, choice of second-line agent may have limited short-term cognitive impact.

In contrast to cognitive outcomes, meta-analytic evidence shows an inverse relationship between sulfonylureas and the risk of ALS (OR = 0.79, 95% CI 0.71–0.89), particularly with longer medication duration (133). However, one included study reported no significant association between insulin use and subsequent ALS onset (OR = 0.80, 95% CI 0.61–1.05). Interpreting the role of diabetes treatment in ALS is confounded by evidence that diabetes itself - particularly non–insulin-dependent diabetes - is associated with lower ALS incidence, making it difficult to disentangle disease-related from drug-specific effects. The lack of a protective association among insulin-treated individuals is consistent with prior findings that insulin-dependent diabetes does not share this inverse relationship with ALS risk.

### 3.3 Advancing diagnosis and monitoring of diabetes and neurological disease

The coexistence of diabetes and neurological disease presents substantial diagnostic complexity. For instance, CIDP represents the most common and treatable inflammatory neuropathy that can be misattributed to diabetes-related peripheral neuropathy. Promisingly, emerging diagnostic tools may be leveraged to resolve this gap: for instance, a 2024 study showed that nerve ultrasound had sensitivity of 87.1% and specificity of 81.9% in detecting CIDP in patients with diabetes (144). Novel electrophysiological techniques such as nerve excitability testing and quantitative sensory testing show promise, detecting subclinical and potentially reversible neuropathic changes with the benefit of portability and progressively decreasing costs (4, 145–148).

Recognizing the interplay between diabetes and cognition, the American Diabetes Association recommends screening for early detection of mild cognitive impairment or dementia for adults 65 years of age or older at the initial visit, annually, and as appropriate (149). Identifying cognitive impairment early has important implications for diabetes care, as cognitive dysfunction makes it challenging to perform complex self-care tasks such as monitoring glucose, administering and adjusting insulin doses, and maintaining appropriate timing and nutritional content of meals. These can increase the risk of hypoglycemia, worsening cognitive function in a vicious cycle. In this context, expert consensus supports simplifying medication regimens and adopting more lenient glycemic targets to optimize diabetes control while minimizing hypoglycemia (150).

Beyond peripheral neuropathy and cognitive impairment, there are no disease-specific guidelines for managing diabetes in the context of neurological disorders. This gap is particularly notable for Parkinson’s Disease, where the relationship with diabetes is well established and influences disease risk, symptom progression, and outcomes. Similarly, for those with diabetes and MS, vision is an essential consideration due to overlapping and potentially irreversible ophthalmic complications, including diabetes-related retinopathy, macular oedema, and MS-related optic neuritis. With immobility being both a common feature of neurological disorders and a principal risk factor of developing DM and metabolic syndrome, metabolic health is a principal concern in patients living with neurological disease and becomes a key component of multidisciplinary care. As neurological-metabolic overlap becomes increasingly recognized, clinical practice will need to evolve to address these co-occurring conditions more explicitly.

Concomitantly, advances in blood-based biomarkers and multi-omic technologies are opening new possibilities for individualized risk stratification and mechanistic disease phenotyping. Among candidate biomarkers, neurofilament light chain (NfL), an intracellular protein released in neurodegeneration, and the astrocytic glial fibrillary acidic protein (GFAP) demonstrate the strongest evidence for predicting incident cognitive impairment in people with diabetes. In the Look AHEAD study of 758 participants with T2DM and overweight or obesity, increasing plasma NfL and GFAP levels over 8–12 years were associated with worsening cognitive function and incident cognitive impairment, whereas baseline levels and changes in amyloid-*β* 42/40 ratio or phosphorylated tau-181 (pTau-181) showed no such associations (151). These non-specific markers of cellular injury are also proposed as strategies for monitoring MS and its progression (152). Other clinically promising biomarkers of neurodegenerative and neurovascular complications in DM are high-sensitivity C-reactive protein, gamma glutamyl transferase (GGT), homocysteine and miRNAs (153). For Parkinsonian syndromes, circulating β-synuclein and UCH-L1 serve as nonspecific measures of neuronal injury (154), but their utility in stratifying PD risk in diabetes remains uncertain.

Novel multi-omic approaches integrating genomics, transcriptomics, proteomics, and metabolomics also show promise. Analysis from human tissues and *in vitro* models reveal convergent metabolic signatures - impaired energy metabolism, oxidative stress, neuroinflammation, endocrine dysregulation, and gut-brain axis alterations - that may underlie diabetes-associated brain vulnerability (155). Polygenic risk scores for neurodegeneration and T2DM show emerging potential, particularly through genes such as APOE, PICALM, SORL1, and GSK3B, although current predictive performance for diabetes remains limited and population-specific (156). Their future value may lie in stratifying individuals with diabetes according to neurological risk, disease severity, and treatment responsiveness.

Ultimately, multi-marker panels integrating neurodegenerative, inflammatory, and metabolic biomarkers, combined with multi-omic and clinical risk data, will likely be required to achieve meaningful diagnostic and prognostic precision. Although considerable challenges remain before these tools can be widely implemented, rapid technological progress suggests that precision neurological risk profiling in diabetes may soon be a tangible reality.

## 4 Future directions

There are several important limitations that confound interpretation of the current evidence linking DM to these neurological disorders. The largely observational nature of the literature precludes conclusions about causal relationships between DM and the named neurological conditions. Diagnostic criteria for many included neurological disorders, e.g., CIDP, are both heterogeneous and evolving, further limiting generalizability. Studies also variably report diabetes-related clinical parameters, including disease duration, glycemic control, treatment exposure, and comorbidities, and frequently do not distinguish between diabetes subtypes, contributing to inconsistencies. This is largely because the existing literature is primarily observational and characterizes DM as a demographic factor alone.

The available evidence also indicates a fundamental distinction between the influence of diabetes on the onset, versus progression, of neurological disease. While diabetes and metabolic dysfunction may increase the risk of incident neurodegenerative disease, emerging evidence suggests a paradoxical relationship during established disease. Higher body mass index has been associated with slower progression and improved survival in ALS, and similar patterns may exist in AD (11). These observations underscore the need to elucidate the underlying pathophysiological mechanisms and highlight the importance of personalized metabolic management influenced by disease stage (for example, less stringent glycemic targets in patients with diabetes and ALS).

This Review highlights several translational implications. Longitudinal, prospective studies are required to clarify causal pathways linking DM and neurological disease. Integrating blood-based biomarkers (e.g., neurofilament light chain, GFAP), advanced electrophysiology, neuroimaging, and multi-omic profiling into well-phenotyped cohorts will enable more precise risk stratification and mechanistic insight into diabetes-associated neurological vulnerability. Crucially, clinical trials of diabetes-related therapies must explicitly evaluate neurological outcomes, particularly for promising agents such as GLP-1RA, SGLT-2 inhibitors, and metformin. Collectively, these efforts have the potential to transform evidence-based guidelines and advance a precision medicine framework at the intersection of diabetes and neurology.

## 5 Conclusion

Diabetes and neurological disease are converging epidemics, and the evidence presented in this Review demonstrates that diabetes intersects with neurodegenerative and neuro-inflammatory conditions in distinct and clinically meaningful ways.

Across neurodegenerative diseases, diabetes is most consistently associated with increased risk and accelerated progression in Alzheimer’s disease, vascular dementia, and Parkinson’s disease, where insulin resistance, cerebrovascular injury, mitochondrial dysfunction, and chronic inflammation converge to cause neurological dysfunction. In contrast, amyotrophic lateral sclerosis and frontotemporal dementia demonstrate more complex and sometimes paradoxical relationships, wherein metabolic dysregulation may delay disease onset yet exacerbate progression once neurodegeneration is established.

For inflammatory nervous system diseases, the relationship to diabetes is grounded in shared immune susceptibility, additive symptom burden, and treatment-mediated effects. The relationship between diabetes and multiple sclerosis reflects overlapping autoimmune pathways, while its association with chronic inflammatory demyelinating polyradiculoneuropathy remains confounded by diagnostic overlap with diabetic neuropathy. In myasthenia gravis, diabetes is overrepresented, yet evidence suggests that corticosteroid exposure, reduced physical activity, and shared inflammatory signaling may drive much of this risk, particularly for T2DM. In contrast, the association between myasthenia gravis and T1DM is more consistent with shared autoimmune susceptibility, supported by genetic data. For NMOSD, emerging evidence suggests that disease-related pathophysiology (notably, IL-6) and treatment-related factors may predispose patients to diabetes. Though evidence is largely derived from small observational studies and preclinical models, it provides a compelling foundation for future research.

Importantly, this Review highlights that diabetes management itself represents a modifiable lever for neurological risk. Lifestyle interventions and glycemic stability emerge as foundational neuroprotective strategies, capable of attenuating inflammation, improving cerebrovascular health, and preserving neuronal energy homeostasis. Beyond this, growing observational and preclinical evidence suggests that specific antidiabetic drug classes, particularly metformin, GLP-1 receptor agonists, and SGLT-2 inhibitors, may exert multifaceted effects on neurodegenerative and neuroinflammatory pathways. While these findings raise the prospect of therapeutic repurposing, current clinical trial data remain limited and, in some cases, conflicting, underscoring the need for rigorously designed, disease-specific trials with neurological endpoints.

Overall, diabetes should be conceptualized as an active biological modifier of neurological disease risk, phenotype, and trajectory. Appreciating this complexity creates opportunities for earlier risk stratification, personalized therapeutic decision-making, and the rational repurposing of metabolic interventions to address neurological health. Recognizing and addressing the metabolic-neurological interface offers a tangible opportunity to integrate clinical management of these conditions and improve outcomes for the increasing number of individuals living with both diabetes and neurological disease worldwide.
